# Association of Epigenetic Metrics of Biological Age With Cortical Thickness

**DOI:** 10.1001/jamanetworkopen.2020.15428

**Published:** 2020-09-14

**Authors:** Amy L. Proskovec, Michael T. Rezich, Jennifer O’Neill, Brenda Morsey, Tina Wang, Trey Ideker, Susan Swindells, Howard S. Fox, Tony W. Wilson

**Affiliations:** 1 Center for Magnetoencephalography, University of Nebraska Medical Center, Omaha; 2 Department of Neurological Sciences, University of Nebraska Medical Center, Omaha; 3 Department of Psychology, University of Nebraska Omaha, Omaha; 4 Magnetoencephalography Center of Excellence, University of Texas Southwestern Medical Center, Dallas; 5 Department of Internal Medicine, Division of Infectious Diseases, University of Nebraska Medical Center, Omaha; 6 Department of Medicine, University of California San Diego, La Jolla; 7 Cognitive Neuroscience of Development & Aging Center, University of Nebraska Medical Center, Omaha

## Abstract

**Importance:**

Magnetic resonance imaging (MRI) studies of aging adults have shown substantial intersubject variability across various brain metrics, and some of this variability is likely attributable to chronological age being an imprecise measure of age-related change. Accurately quantifying one’s biological age could allow better quantification of healthy and pathological changes in the aging brain.

**Objective:**

To investigate the association of DNA methylation (DNAm)–based biological age with cortical thickness and to assess whether biological age acceleration compared with chronological age captures unique variance in cortical thinning.

**Design, Setting, and Participants:**

This cross-sectional study used high-resolution structural brain MRI data collected from a sample of healthy aging adults who were participating in a larger ongoing neuroimaging study that began in May 2014. This population-based study accrued participants from the greater Omaha, Nebraska, metropolitan area. One hundred sixty healthy adults were contacted for the MRI component, 82 of whom participated in both DNAm and MRI study components. Data analysis was performed from March to June 2019.

**Main Outcomes and Measures:**

Vertexwise cortical thickness, DNAm-based biological age, and biological age acceleration compared with chronological age were measured. A pair of multivariable regression models were computed in which cortical thickness was regressed on DNAm-based biological age, controlling for sex in the first model and also controlling for chronological age in the second model.

**Results:**

Seventy-nine adult participants (38 women; mean [SD] age, 43.82 [14.50] years; age range, 22-72 years) were included in all final analyses. Advancing biological age was correlated with cortical thinning across frontal, superior temporal, inferior parietal, and medial occipital regions. In addition, biological age acceleration relative to chronological age was associated with cortical thinning in orbitofrontal, superior and inferior temporal, somatosensory, parahippocampal, and fusiform regions. Specifically, for every 1 year of biological age acceleration, cortical thickness would be expected to decrease by 0.024 mm (95% CI, −0.04 to −0.01 mm) in the left orbitofrontal cortex (partial *r*, −0.34; *P* = .002), 0.014 mm (95% CI, −0.02 to −0.01 mm) in the left superior temporal gyrus (partial *r*, −0.36; *P* = .001), 0.015 mm (95% CI, −0.02 to −0.01 mm) in the left fusiform gyrus (partial *r*, −0.38; *P* = .001), 0.015 mm (95% CI, −0.02 to −0.01 mm) in the right fusiform gyrus (partial *r*, −0.43; *P* < .001), 0.019 mm (95% CI, −0.03 to −0.01 mm) in the right inferior temporal sulcus (partial *r*, −0.34; *P* = .002), and 0.011 mm (95% CI, −0.02 to −0.01 mm) in the right primary somatosensory cortex (partial *r*, −0.37; *P* = .001).

**Conclusions and Relevance:**

To our knowledge, this is the first study to investigate vertexwise cortical thickness in relation to DNAm-based biological age, and the findings suggest that this metric of biological age may yield additional insight on healthy and pathological cortical aging compared with standard measures of chronological age alone.

## Key Points

**Question:**

Are DNA methylation–based metrics of biological age associated with cortical thickness, and does a biological age acceleration index capture unique changes in cortical thinning compared with chronological age measures?

**Findings:**

In this cross-sectional study of 82 healthy adults, both biological and chronological age were associated with widespread cortical thinning, but biological age acceleration was also associated with additional changes in cortical morphological features. Older biological age relative to chronological age was associated with accentuated thinning within prefrontal and temporal regions.

**Meaning:**

These findings suggest that DNA methylation–based biological age may yield additional insight into healthy and pathological cortical aging compared with chronological age alone.

## Teaser

This cross-sectional study investigates the association of DNA methylation–based biological age with cortical thickness and assesses whether a biological age acceleration index compared with chronological age captures unique variance in cortical thinning.

## Introduction

It is well established that advancing age is accompanied by changes in brain morphological features, and among these changes are age-related reductions in cortical thickness. In healthy adults, age-related thinning is observed across the majority of the cortex but is generally more robust in bilateral frontal cortices, superior temporal regions, and supramarginal gyri.^1,2,3^ Despite these overarching patterns, substantial variability exists among individuals, with some older adults demonstrating preserved cortical structure, whereas others are characterized by accentuated cortical thinning.^2^ Indeed, such individual variability suggests that chronological age is an imperfect indicator of the rate of neurostructural decline and highlights the importance of identifying biomarkers that can reliably estimate healthy and pathological brain aging. Epigenetic markers of aging may prove fruitful in this regard.

Recently, one epigenetic marker that has gained traction is DNA methylation (DNAm), in which the presence of methylated cytosine at cytosine-phosphate-guanine (CpG) sites is evaluated.^4^ Previous research has demonstrated that DNAm levels change with age.^4^ In fact, the methylation levels at certain CpG sites in the genome are so tightly associated with age that they have been used to estimate chronological age with a high degree of accuracy.^5,6^ These sets of CpGs are often referred to as epigenetic clocks, and arguably the 2 most widely used clocks are those of Hannum et al,^5^ which is based on the methylation levels of 71 CpGs, and Horvath,^6^ which incorporates 353 CpG sites. The age estimate that these clocks produce is frequently called *epigenetic age* or *DNAm age*.^7^ Because DNAm is thought to index biological age, discrepancies between methylation-based age and chronological age are indicative of accelerated or decelerated biological aging, and such measures of relative biological age acceleration are highly heritable.^6^ Importantly, relative biological age acceleration has been associated with various health conditions, including Alzheimer disease,^8^ Parkinson disease,^9^ Huntington disease,^10^ Down syndrome,^11^ and HIV,^12^ and is associated with cancer,^13,14,15^ cardiovascular disease,^15^ and all-cause mortality.^15,16,17^

Although the aforementioned research reinforces the exciting possibility that DNAm age and relative biological age acceleration provide superior utility to estimate disease and respective risk compared with chronological age alone, only a handful of studies have begun to characterize the association of relative biological age acceleration with brain-based morphological characteristics. Specifically, relative biological age acceleration has been associated with decreased global white matter integrity^18^ and increased white matter hyperintensity burden and severity^19^ in healthy adults. Relative biological age acceleration has been associated with degraded neural integrity in the genu of the corpus callosum in veterans, most of whom were diagnosed with posttraumatic stress disorder,^20^ and has been positively associated with fractional anisotropy and negatively associated with mean diffusivity in a sample of adults, half of whom presented with mild cognitive impairment.^21^ Finally, in a sample of adolescent girls, relative biological age acceleration was negatively associated with left hippocampal volume.^22^ These findings suggest that relative biological age acceleration may indeed be a viable biomarker of brain aging. However, much remains to be determined, and whether relative biological age acceleration is associated with cortical volumetrics is currently unknown. Because evidence suggests that cortical thickness is more sensitive to age-related effects than gray matter volume^23^ and that abnormal patterns of cortical thinning are observed in age-related pathological processes (eg, Alzheimer disease),^2,24^ characterizing the association of relative biological age acceleration with cortical thickness may lead to new discoveries, as well as a more accurate method of estimating deleterious changes in cortical morphological features and the risk of associated age-related diseases.

The present study takes the first step toward actualizing this goal. Here, we used 2 established measures of DNAm-based biological age and surface-based morphometry to investigate the association of biological age acceleration with cortical thickness in healthy adults. We hypothesized that, similar to chronological age, DNAm age would be negatively associated with cortical thickness in bilateral frontal, superior temporal, and inferior parietal cortices. We also posited that biological age acceleration would be uniquely associated with cortical thinning, beyond the effects of chronological age.

## Methods

### Subject Selection

After providing a complete description of the study, written informed consent was obtained from participants following the guidelines of the University of Nebraska Medical Center’s institutional review board, which approved the study protocol. This cross-sectional study follows the Strengthening the Reporting of Observational Studies in Epidemiology (STROBE) reporting guideline.

We recruited 82 healthy adults (40 women; mean [SD] age, 44.21 [14.88] years; age range, 22-72 years) from the local community to be part of a larger neuroimaging study conducted from May 2014 onward. The data reported here were collected between May 2014 and September 2017. Exclusion criteria included any medical illness affecting central nervous system function, neurological or psychiatric disorder, history of head trauma, cognitive impairment, current substance abuse, and ferromagnetic implants.

### Blood Sampling and Methylation Analysis

Whole-blood samples were obtained from each participant as closely as possible to their magnetic resonance imaging (MRI) scan date. All analyses involving DNAm metrics were run both with and without including the difference between these 2 time points as a covariate of no interest. Importantly, the results were virtually identical, and all statistically significant findings held, irrespective of whether this covariate was included or excluded in the models. Thus, the results reported here are from the models in which the number of days between the blood sampling and the MRI scan was not included as a covariate. The DNA sample collection, methylation analysis, and DNAm age estimation closely followed the pipeline established in earlier work.^12^ Briefly, DNA was purified from whole-blood samples using EDTA collection tubes (Vacutainer; BD) and blood and tissue extraction kits (DNeasy; Qiagen). Methylation analysis was performed using Infinium HumanMethylation450 BeadChip Kits (Illumina). After hybridization, BeadChips were scanned using the Illumina HiScan System. All data were processed through the Minfi R processing pipeline.^25^ Methylome data were downloaded from Hannum et al^5^ and EPIC^26^ (Gene Expression Omnibus, GSE40279 and GSE51032) and were processed alongside the methylation data generated from our sample. Beta values were extracted and quantile normalized using Minfi, cell counts were estimated using estimate Cell Composition, and the resulting normalized beta values were adjusted for cell types.^12,27^ All data were then normalized using a modified BMIQ procedure provided by Horvath.^6^ The reference standard was set to the median beta observed in the study by Hannum et al.^5^

The consensus model of DNAm age was used for all analyses, which amalgamates the estimation methods of both Hannum et al^5^ and Horvath.^6^ Consensus DNAm age has previously been found to harbor superior estimating capacity compared with either model in isolation.^12^ Following convention,^12,19^ the residuals from regressing DNAm age on chronological age at the time of blood collection were used to quantify biological age acceleration relative to chronological age. Calculation of relative biological age acceleration revealed 2 outliers who had values that were beyond 3 SD away from the mean and were excluded from subsequent analyses.

### Structural MRI Acquisition and Processing

All participants underwent high-resolution, T1-weighted MRI on a 3-T scanner (Achieve X-series; Philips Healthcare) using an 8-channel head coil and a 3D fast-field echo sequence (repetition time, 8.09 ms; echo time, 3.7 ms; field of view, 240 mm; slice thickness, 1.0 mm with no gap; in-plane resolution, 1.0 × 1.0 mm). The structural MRI data were processed using the standard pipeline in the CAT12 toolbox version 12.6 (Jena University Hospital) at a resolution of 1 mm^3^ within SPM software version 12 (Wellcome Trust Centre for Neuroimaging) using MATLAB statistical software version 2017b (MathWorks). The surface-based morphometry pipeline in CAT12 is fully automated and uses a projection-based thickness approach to estimate cortical thickness and reconstruct the central surface in 1 step.^28^ Essentially, after tissue segmentation,^29^ the white matter distance is estimated, and the local maxima (which is equal to the cortical thickness) are projected onto other gray matter voxels using a neighboring relationship described by the white matter distance. Projection-based thickness accounts for partial volume correction, sulcal blurring, and sulcal asymmetries without sulcus reconstruction. To rectify topological defects, a correction based on spherical harmonics was used,^30^ and the cortical surface mesh was reparameterized into a common coordinate system via an algorithm that reduces area distortion.^31^ Finally, the resulting maps were resampled and smoothed using a 15-mm, full-width, half-maximum gaussian kernel.

For quality assurance, a 2-step process was adopted. First, before segmentation, data were visually inspected for artifacts, and 1 scan was excluded from subsequent statistical analyses because of excessive motion-related artifacts in the T1-weighted data. Second, the quality control measures incorporated in the CAT12 processing pipeline were used to identify the most deviant data after segmentation. These data were inspected further for the presence of newly introduced artifacts.

### Statistical Analyses

To examine the association of biological age metrics with cortical thickness, we used the general linear model offered in CAT12 and SPM12 to perform vertexwise analyses. Specifically, to investigate the association of biological age with cortical thickness, we regressed the cortical thickness data on DNAm age and included sex as a covariate of no interest. To characterize the association of relative biological age acceleration with cortical thickness, cortical thickness was regressed on DNAm age, with sex and chronological age at the time of blood sampling included as covariates of no interest. All output statistical maps were displayed as a function of α level, thresholded at 2-sided *P* < .01, and adjusted for multiple comparisons using a spatial extent threshold (ie, cluster restriction) based on the theory of gaussian random fields.^32,33^ The cluster extent thresholds applied were empirically determined for each statistical test, and the final results were corrected for nonisotropic smoothness. Of note, we also conducted nonparametric permutation testing using threshold-free cluster enhancement^34^ with the number of permutations set at 5000. These statistical parametric maps were thresholded at 2-sided *P* < .05, and all of our results survived this additional control for type I error. Data analysis was performed from March to June 2019.

## Results

### Association of DNAm Age With Chronological Age

**Figure 1.**
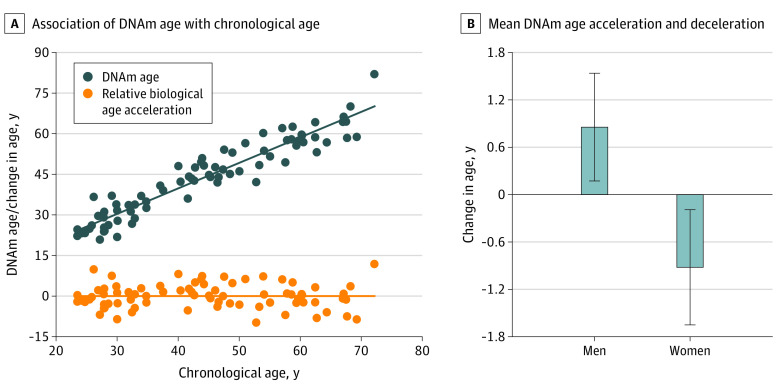
Association of DNA Methylation (DNAm) Age and Chronological Age Panel A shows a scatterplot displaying the association of chronological age with DNAm-based biological age (blue dots), with the line of best fit overlaid. Additionally, the plot depicts the biological age acceleration or deceleration (relative biological age acceleration) for each participant (orange dots). Panel B shows a bar graph depicting the mean number of years biological age was accelerated or decelerated relative to chronological age in men and women. Error bars show the standard error of the mean.

Seventy-nine adult participants (38 women) spanning a wide chronological age range (mean [SD] age, 43.82 [14.50] years; age range 22-72 years) were included in all final analyses. DNAm age was significantly associated with chronological age (mean [SE] *b*, 0.96 [0.04]; 95 CI, 0.89-1.03; β = 0.95; *t*_77_ = 26.45; *R^2^* = 0.90; adjusted *R^2^* = 0.90; *P* < .001) (Figure 1). The residuals from regressing DNAm age on chronological age were used as a measure of biological age acceleration (ie, relative biological age acceleration), and ranged from −9.76 to 11.86 years (mean [SD], 0.0 [4.49] years; median, 0.03 years; interquartile range, −2.64 to 2.77 years). Because previous research has demonstrated differences in relative biological age acceleration between male and female individuals,^5,7,35^ we probed whether such sex differences replicated in our sample. Women typically had younger biological age relative to chronological age (mean [SD], −0.92 [4.50] years; median, −1.32 years; interquartile range, −4.06 to 2.10 years), whereas men generally had older biological age relative to chronological age (mean [SD], 0.85 [4.37] years; median, 0.60 years; interquartile range, −1.85 to 3.67 years), but the difference was not significant (*t*_77_ = 1.78; *P* = .08) (Figure 1).

### Association of DNAm Age With Cortical Thickness

**Figure 2.**
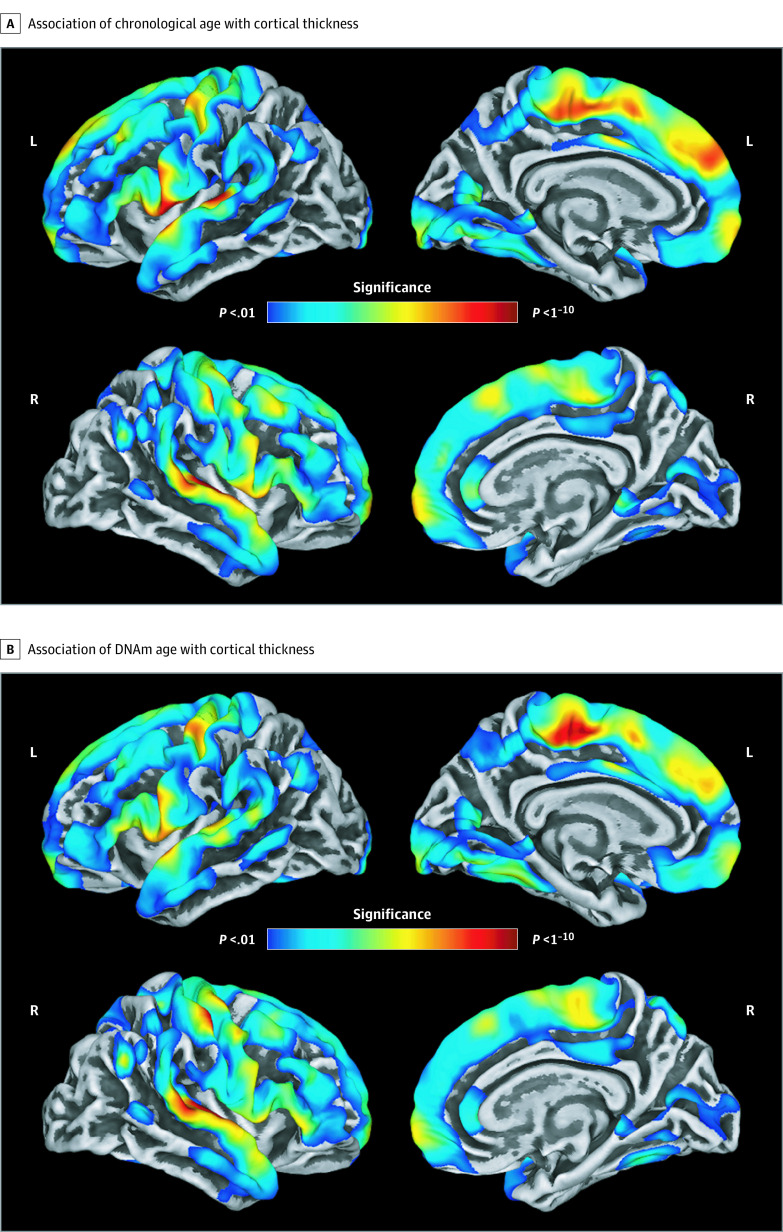
Association of Chronological Age and DNA Methylation (DNAm) Age With Cortical Thickness Maps show vertexwise regressions of cortical thickness on chronological age (A) and DNAm-based biological age (B), controlling for sex. Both chronological age and biological age were significantly associated with cortical thickness across widespread frontal, superior temporal, inferior parietal, and medial occipital regions (all *P* < .001, corrected). In all regions detected, advancing age was associated with reductions in cortical thickness. In both panels, the top left image shows the lateral view of the left hemisphere, the top right image shows the medial view of the left hemisphere, the bottom left image shows the lateral view of the right hemisphere, and the bottom right image shows the medial view of the right hemisphere.

To examine the associations of cortical thickness with chronological and biological age, vertexwise cortical thickness was regressed on chronological or biological age, respectively, while controlling for sex. Both analyses yielded a very similar pattern of results. Significant negative associations were observed between each age metric (ie, chronological age and DNAm age) and cortical thickness across widespread frontal regions and superior temporal, primary motor, primary somatosensory, inferior parietal, and medial occipital cortices (Figure 2). That is, as chronological age or biological age increased, cortical thickness within these regions decreased, and these associations were significant in bilateral superior temporal, inferior parietal, primary somatosensory, primary motor, and supplementary motor cortices. Additionally, chronological and biological age were both significantly and negatively associated with global cortical thickness (see eAppendix and eTable 1 in the Supplement).

### Association of DNAm Age Acceleration With Cortical Thickness

**Figure 3.**
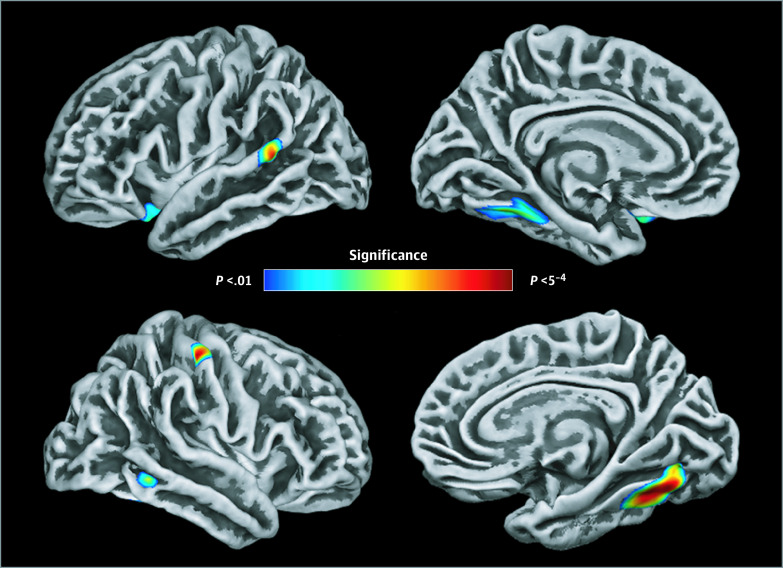
Association of DNA Methylation (DNAm) Age Acceleration or Deceleration With Cortical Thickness Maps show vertexwise regressions of cortical thickness on DNAm-based biological age, controlling for sex and chronological age. That is, these maps depict the association of biological age acceleration or deceleration with cortical thickness. Greater biological age acceleration was associated with greater cortical thinning within left orbitofrontal, superior temporal, and parahippocampal regions, right inferior temporal and somatosensory cortices, and bilateral fusiform regions (*P* < .01, corrected). The top left image shows the lateral view of the left hemisphere, the top right image shows the medial view of the left hemisphere, the bottom left image shows the lateral view of the right hemisphere, and the bottom right image shows the medial view of the right hemisphere.

**Figure 4.**
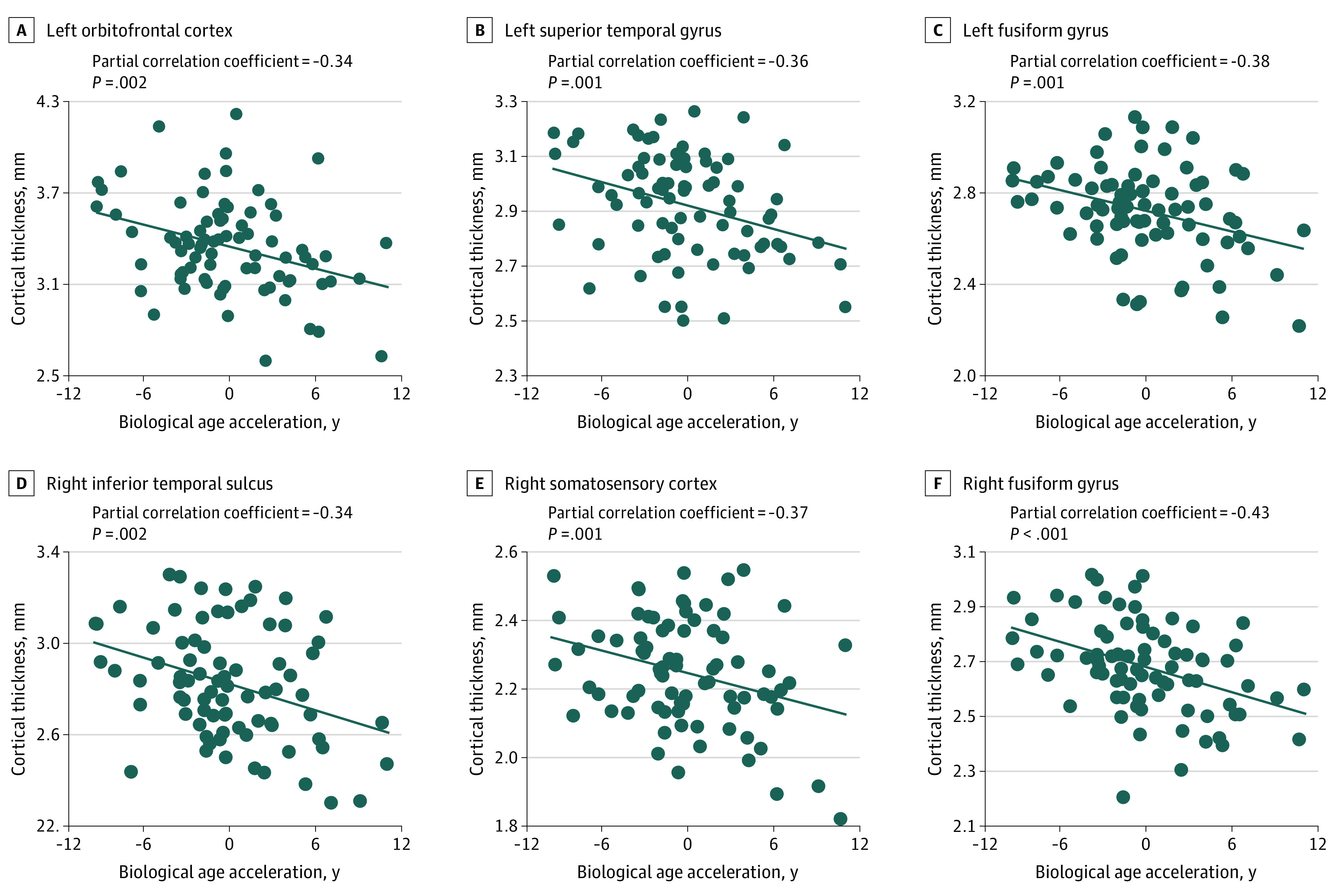
Associations of DNA Methylation (DNAm) Age Acceleration With Cortical Thickness Scatterplots show the associations of biological age acceleration and deceleration with cortical thickness for each neural region in which associations were observed (shown in Figure 3).

To determine whether biological age was uniquely associated with cortical thickness, above and beyond the effect of chronological age, vertexwise cortical thickness was regressed on DNAm age while controlling for chronological age and sex. In other words, this model tested whether biological age acceleration relative to chronological age was associated with cortical thickness. The results indicated significant and negative correlations between relative biological age acceleration and the cortical thickness of the left orbitofrontal cortex, posterior superior temporal gyrus, and left parahippocampal gyrus extending into the fusiform gyrus, as well as the right inferior temporal sulcus, primary somatosensory cortex, and fusiform gyrus (Figure 3). That is, persons with greater biological age acceleration (ie, older biological relative to chronological age) had more pronounced cortical thinning within these brain regions. Specifically, for every 1 year of biological age acceleration, cortical thickness would be expected to decrease by 0.024 mm (95% CI, −0.04 to −0.01 mm) in the left orbitofrontal cortex (partial *r*, −0.34; *P* = .002), 0.014 mm (95% CI, −0.02 to −0.01 mm) in the left superior temporal gyrus (partial *r*, −0.36; *P* = .001), 0.015 mm (95% CI, −0.02 to −0.01 mm) in the left fusiform gyrus (partial *r*, −0.38; *P* = .001), 0.015 mm (95% CI, −0.02 to −0.01 mm) in the right fusiform gyrus (partial *r*, −0.43; *P* < .001), 0.019 mm (95% CI, −0.03 to −0.01 mm) in the right inferior temporal sulcus (partial *r*, −0.34; *P* = .002), and 0.011 mm (95% CI, −0.02 to −0.01 mm) in the right primary somatosensory cortex (partial *r*, −0.37; *P* = .001) (Figure 4 and Table; see eTable 2 in the Supplement for overall model statistics). As mentioned in the Methods section, we also applied threshold-free cluster enhancement to correct for multiple comparisons; these results were very similar and are included in the eFigure in the Supplement. Finally, in a follow-up analysis, we observed that individuals who had thinner cortex in 1 region had thinner cortex in each other region (see eAppendix and eTable 3 in the Supplement).

**Table.** Hierarchical Multiple Regression Results for Cortical Thickness on Relative Biological Age Acceleration or Deceleration While Controlling for Sex^a^

Region	*b* (SE) [95% CI], mm	*t*_3,75_	*P* value	β	*F* change	*R*^2^ change	*f*^2^	Partial *r*
Left								
Orbitofrontal cortex	−0.024 (0.008) [−0.04 to −0.01]	−3.15	.002	−1.10	9.94	0.12	0.13	−0.34
Superior temporal gyrus	−0.014 (0.004) [−0.02 to −0.01]	−3.39	.001	−1.08	11.47	0.11	0.15	−0.36
Fusiform gyrus	−0.015 (0.004) [−0.02 to −0.01]	−3.55	.001	−1.10	12.60	0.12	0.17	−0.38
Right								
Inferior temporal sulcus	−0.019 (0.006) [−0.03 to −0.01]	−3.17	.002	−1.10	10.03	0.12	0.13	−0.34
Primary somatosensory cortex	−0.011 (0.003) [−0.02 to −0.01]	−3.40	.001	−0.99	11.58	0.09	0.15	−0.37
Fusiform gyrus	−0.015 (0.004) [−0.02 to −0.01]	−4.09	<.001	−1.32	16.69	0.17	0.22	−0.43

Abbreviations: *b*, unstandardized regression coefficient; β, standardized regression coefficient; *f*^2^, Cohen local effect size.

^a^ Data are shown for 79 patients.

## Discussion

In this study, we used surface-based morphometry and blood-based DNAm to characterize the association of biological age acceleration with cortical thickness in a large sample of healthy individuals whose age range extended across most of the adult human life span. Similar to advancing chronological age, advancing biological age was associated with widespread cortical thinning in frontal, superior temporal, inferior parietal, and medial occipital regions. In addition, greater biological age acceleration (ie, older biological relative to chronological age) was associated with accentuated thinning within the prefrontal, superior temporal, inferior temporal, somatosensory, parahippocampal, and fusiform cortices.

The pattern of cortical thinning associated with advancing biological age closely paralleled that of chronological age advancement and aligned with the results of prior studies^1,3,23,36^ that investigated the association of chronological age with cortical thickness. Because DNAm epigenetic clocks were designed to be robust estimators of chronological age^5,6^ and given that the biological age estimates computed in the present study demonstrated this association, the highly overlapping patterns of chronological and biological aging associations with cortical thickness were to be expected. Essentially, the present findings provide evidence that DNAm age captured what it should (ie, the age associations with cortical thickness that have been consistently demonstrated in the literature).

What is arguably more exciting is that DNAm age captured additional associations with cortical thickness, above and beyond those identified when using chronological age in isolation. These unique associations with biological age acceleration were observed in cortical regions that have been implicated in sensory and cognitive processes susceptible to age-related decline. For example, the fusiform and ventral temporal cortices are constituents of the ventral visual processing stream (ie, the *what pathway*) and have been associated with object and face recognition.^37,38^ Age-related deficits in facial recognition and, more broadly, visual perception and processing speed are well documented within the literature.^39,40^ Given the association observed here between accelerated biological age and thinning within regions of the ventral visual stream, investigating the associations between these variables and behavioral indices of visual perception should be the target of future research. In a similar fashion, the parahippocampal cortex is robustly implicated in spatial navigation^41^; the left posterior superior temporal cortex is integral to language processes, including the storage and retrieval of phonological information^42^; the orbitofrontal cortex is closely tied to reward processing, emotion, and decision-making^43,44^; and the primary somatosensory cortex underlies tactile sensation and spatial acuity,^45^ all of which are constructs that are known to be susceptible to age-related change.^46,47,48,49,50,51,52,53,54,55^ Taken together, these findings present promising areas for further functional investigations in future studies.

### Limitations

It is important to recognize limitations of the present study. First, DNAm was evaluated in peripheral blood, rather than the organ of interest (ie, brain), and age-related differences in DNAm have been shown across some tissue types.^56,57^ Thus, DNAm age estimates derived from cortical tissue may prove to be more accurate factors associated with change in cortical morphological features. However, remarkable consistency regarding age effects on DNAm levels has been demonstrated across various cell and tissue types, with some of the greatest consonance observed between blood and brain tissue.^6,58^ Because collection of neural tissue samples from living humans is not feasible, our results in amalgamation with these prior findings suggest that DNAm in blood is an accessible and viable biomarker of brain aging. A second limitation was the cross-sectional design of the present study. As such, our results specifically capture associations with age, rather than aging. In addition, evidence suggests that a cross-sectional approach may underestimate age-related effects on brain morphological features compared with a longitudinal approach. Thus, adopting a longitudinal design in future research is an important consideration.

## Conclusions

To our knowledge, the present study is the first to investigate the association of DNAm-based biological age with vertexwise cortical thickness. The data indicate that biological age not only captures the expected age-related associations with cortical thickness, but uniquely highlights additional changes in cortical morphological features. As such, DNAm age and biological age acceleration may indeed provide greater insight on healthy and pathological cortical aging, compared with chronological age alone. On a broader scale, our data also align with the growing literature that supports the use of DNAm as a viable biomarker of aging. In addition to eliminating some of the variability seen with chronologically based age metrics, using a more precise measure of biological age could aid in the early detection of disorders, possibly before clinical symptoms manifest, as well as in elucidating risk factors (eg, obesity) for various health conditions. This is a particularly exciting prospect, as evidence suggests that epigenetic changes are reversible,^7^ which further motivates investigation into the use of DNAm age in the assessment of well-being. Finally, although adult aging was the focus of the current investigation, DNAm age may also illuminate developmental trajectories more precisely, because it is likely to capture puberty onset more accurately than chronological age.

## Notes

Supplement. **eAppendix.** Follow-up Analyses **eTable 1.** Hierarchical Multiple Regression Results for Global Cortical Thickness on Chronological Age, DNAm Age, and DNAm Age Acceleration/Deceleration While Controlling for Sex **eTable 2. **Overall Model Summary for the Hierarchical Multiple Regression of Cortical Thickness on DNAm Age Acceleration/Deceleration While Controlling for Sex **eFigure.** Relationship Between DNAm Age Acceleration/Deceleration and Cortical Thickness When Using Threshold-Free Cluster Enhancement to Correct for Multiple Comparisons **eTable 3.** Pearson Correlation Coefficients Between the Cortical Thickness of Each Neural Region in Which DNAm Age Acceleration/Deceleration Associations Were Observed
