# Supplementary material for: Association of Epigenetic Metrics of Biological Age With Cortical Thickness
Source: JAMA Netw Open. 2020 Sep 14;3(9):e2015428. doi: 10.1001/jamanetworkopen.2020.15428 (PMC7490648; doi:10.1001/jamanetworkopen.2020.15428)
Supplement: Supplement. — eAppendix. Follow-up Analyses eTable 1. Hierarchical Multiple Regression Results for Global Cortical Thickness on Chronological Age, DNAm Age, and DNAm Age Acceleration/Deceleration While Controlling for Sex eTable 2. Overall Model Summary for the Hierarchical Multiple Regression of Cortical Thickness on DNAm Age Acceleration/Deceleration While Controlling for Sex eFigure. Relationship Between DNAm Age Acceleration/Deceleration and Cortical Thickness When Using Threshold-Free Cluster Enhancement to Correct for Multiple Comparisons eTable 3. Pearson Correlation Coefficients Between the Cortical Thickness of Each Neural Region in Which DNAm Age Acceleration/Deceleration Associations Were Observed [file jamanetwopen-e2015428-s001.pdf]

Supplementary Online Content

Proskovec AL, Rezich MT, O'Neill J, et al. Association of epigenetic metrics of biological age with cortical thickness. *JAMA Netw Open*. 2020;3(9):e2015428. doi:10.1001/jamanetworkopen.2020.15428

eAppendix. Follow-up Analyses
eTable 1. Hierarchical Multiple Regression Results for Global Cortical Thickness on Chronological Age, DNAm Age, and DNAm Age Acceleration/Deceleration While Controlling for Sex
eTable 2. Overall Model Summary for the Hierarchical Multiple Regression of Cortical Thickness on DNAm Age Acceleration/Deceleration While Controlling for Sex
eFigure. Relationship Between DNAm Age Acceleration/Deceleration and Cortical Thickness When Using Threshold-Free Cluster Enhancement to Correct for Multiple Comparisons
eTable 3. Pearson Correlation Coefficients Between the Cortical Thickness of Each Neural Region in Which DNAm Age Acceleration/Deceleration Associations Were Observed

This supplementary material has been provided by the authors to give readers additional information about their work.

© 2020 Proskovec AL et al. *JAMA Network Open.*

eAppendix. Follow-up Analyses

As a follow-up analysis, we examined the association of global cortical thickness with chronological and biological age by regressing global cortical thickness on chronological age or DNAm age, respectively, while controlling for sex. Chronological age was significantly and negatively related to global cortical thickness, $\Delta R^2 = .29$, $p < .001$, such that for every one year increase in chronological age, a .004 mm decrease in global cortical thickness would be expected. Similarly, biological age was significantly and negatively associated with global cortical thickness, $\Delta R^2 = .30$, $p < .001$, such that for every one year increase in biological age, a .004 mm decrease in global cortical thickness would be expected. We also investigated the relationship between ΔAge and global cortical thickness by regressing global cortical thickness on DNAm age while controlling for chronological age and sex. ΔAge was not significantly correlated with global cortical thickness, $\Delta R^2 = .01$, $p = .24$. The full results are displayed in eTable 1, below.

In a second follow-up analysis we investigated whether the same individuals demonstrated reduced cortical thickness in each of the regions in which ΔAge relationships were observed, by computing a series of Pearson correlations between the cortical thickness values of each pair of regions. After applying a Bonferroni correction to control for multiple comparisons, all but two relationships remained significant ($p < .05$, corrected). Those relationships which were not significant were between the right primary somatosensory cortex and left orbitofrontal cortex, and the right inferior temporal sulcus and left orbitofrontal cortex. All relationships were positive, such that individuals who had thinner cortex in one region tended to have thinner cortex in each other region (see eTable 3, below).

eTable 1. Hierarchical Multiple Regression Results for Global Cortical Thickness on Chronological Age, DNAm Age, and DNAm Age Acceleration/Deceleration While Controlling for sex

Age Metric	*b*	*SE*	*t*	*p*	β	*F*	R^2	Δ*F*	ΔR^2	95% CI
Chronological	-.004	.001	-5.61*	<.001	-.55	15.88*	.30	31.51	.29	(-.005, -.003)
DNAm	-.004	.001	-5.74*	<.001	-.55	16.59*	.30	32.92	.30	(-.005, -.003)
ΔAge	-.003	.002	-1.18	.24	-.37	11.10*	.31	1.38	.01	(-.007, .002)

Note. N = 79. ΔAge = DNAm age acceleration/deceleration. b = unstandardized regression coefficient. SE = standard error. β = standardized regression coefficient. ΔF = F change. ΔR^2 = R^2 change. CI = confidence interval. *p < .001.

© 2020 Proskovec AL et al. *JAMA Network Open.*

eTable 2. Overall Model Summary for the Hierarchical Multiple Regression of Cortical Thickness on DNAm Age Acceleration/Deceleration While Controlling for Sex

Region	*F*	*SE*	R^2	aR^2	*p*
L OFC	3.57	.29	.13	.09	.02
L STG	9.68	.16	.28	.25	<.001
L FG	11.24	.17	.31	.28	<.001
R ITS	3.90	.24	.14	.10	.01
R S1	16.06	.13	.39	.38	<.001
R FG	8.81	.15	.26	.23	<.001

Note. N = 79. OFC = orbitofrontal cortex. STG = superior temporal gyrus. FG = fusiform gyrus. ITS = inferior temporal sulcus. S1 = primary somatosensory cortex. *SE* = standard error. aR^2 = adjusted R^2. ΔF = *F* change. ΔR^2 = R^2 change. f^2 = Cohen's local effect size. *pr* = partial correlation coefficient. CI = confidence interval.

© 2020 Proskovec AL et al. *JAMA Network Open.*

eFigure. Relationship Between DNAm Age Acceleration/Deceleration and Cortical Thickness When Using Threshold-Free Cluster Enhancement to Correct for Multiple Comparisons

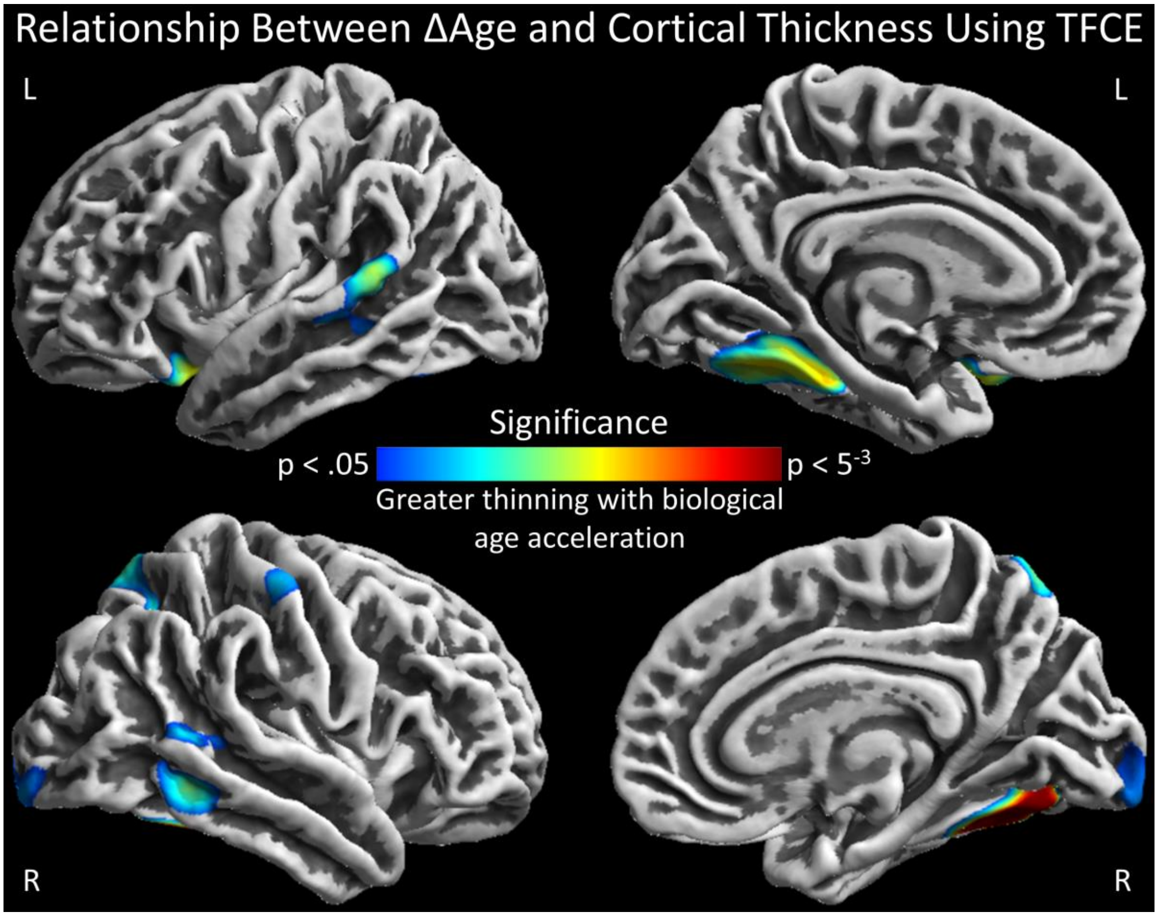

Maps displaying vertex-wise regressions of cortical thickness on methylation-based biological age, controlling for sex and chronological age. That is, these maps depict the relationship between biological age acceleration/deceleration and cortical thickness. Greater biological age acceleration was predictive of greater cortical thinning within left orbitofrontal, superior temporal, and parahippocampal regions, right inferior temporal, temporo-parietal, somatosensory, superior parietal, and primary visual cortices, and bilateral fusiform regions ($p < .05$, corrected).

© 2020 Proskovec AL et al. *JAMA Network Open.*

eTable 3. Pearson Correlation Coefficients Between the Cortical Thickness of Each Neural Region in Which DNAm Age Acceleration/Deceleration Associations Were Observed

	L OFC	L STG	L FG	R ITS	R S1
**L STG**	.34*				
**L LG**	.40*	.55*			
**R ITS**	.22	.55*	.41*		
**R S1**	.14	.49*	.54*	.33*	
**R LG**	.34*	.56*	.76*	.45*	.49*

Note. $N = 79$. OFC = orbitofrontal cortex. STG = superior temporal gyrus. FG = fusiform gyrus. ITS = inferior temporal sulcus. S1 = primary somatosensory cortex. *$p < .05$, Bonferroni corrected.

© 2020 Proskovec AL et al. *JAMA Network Open.*
